# Throughput and resolution with a next-generation direct electron detector

**DOI:** 10.1107/S2052252519012661

**Published:** 2019-10-24

**Authors:** Joshua H. Mendez, Atousa Mehrani, Peter Randolph, Scott Stagg

**Affiliations:** a Department of Physics, Florida State University, 77 Chieftan Way, Tallahassee, FL 32306, USA; b Department of Chemistry and Biochemistry, Florida State University, 95 Chieftain Way, Tallahassee, FL 32306, USA; c Institute of Molecular Biophysics, Florida State University, 91 Chieftain Way, Tallahassee, FL 32306, USA

**Keywords:** 3D reconstruction, image processing, advances in microscope hardware, single-particle cryo-EM, direct electron detectors

## Abstract

Direct electron detectors (DEDs) have revolutionized cryo-electron microscopy (cryo-EM) by facilitating the correction of beam-induced motion and radiation damage, and also by providing high-resolution image capture. A new-generation DED, the DE64, has been developed by Direct Electron that has good performance in both integrating and counting modes. The camera has been characterized in both modes in terms of image quality, throughput and resolution of cryo-EM reconstructions. The modulation transfer function, noise power spectrum and detective quantum efficiency (DQE) were determined for both modes, as well as the number of images per unit time. Although the DQE for counting mode was superior to that for integrating mode, the data-collection throughput for this mode was more than ten times slower. Since throughput and resolution are related in single-particle cryo-EM, data for apoferritin were collected and reconstructed using integrating mode, integrating mode in conjunction with a Volta phase plate (VPP) and counting mode. Only the counting-mode data resulted in a better than 3 Å resolution reconstruction with similar numbers of particles, and this increased performance could not be compensated for by the increased throughput of integrating mode or by the increased low-frequency contrast of integrating mode with the VPP. These data show that the superior image quality provided by counting mode is more important for high-resolution cryo-EM reconstructions than the superior throughput of integrating mode.

## Abstract 2

The superior image quality given by the DE64 direct electron detector in counting mode is more important for high-resolution cryo-EM reconstructions than its superior throughput in integrating mode.

## 1. Introduction   

The technology used in recording images for cryo-electron microscopy (cryo-EM) has evolved from the use of photographic film to charge-coupled devices (CCD) and recently direct electron detector (DED) cameras. The progression of data-collection techniques has allowed the automation of data collection, an increased signal-to-noise ratio (SNR) of images and the correction of beam-induced motion. Direct detectors can operate in one of two modes: integrating mode, in which electron hits deposit some amount of charge that is built up over the course of an exposure, and counting mode, in which individual electron hits are ‘counted’ and the counts are summed over the exposure. Counting mode eliminates the Landau noise that results from individual electrons depositing different amounts of energy on the detector. On the other hand, counting requires a very weak beam and long exposure times, which can result in lower throughput. These detector advancements, together with the development of new software, have helped cryo-EM to break the 3 Å resolution boundary (Herzik *et al.*, 2017 ▸). A new-generation DED called the DE64 built by Direct Electron promises good performance when used in either integrating or counting mode. Resolution in single-particle reconstructions is related to the amount of data that contribute to the reconstruction (Stagg *et al.*, 2014 ▸; Heymann, 2019 ▸); therefore, the question arises of whether it is better to collect faster potentially lower quality data in integrating mode or slower higher quality data in counting mode.

Here, we have characterized the DE64 and compared its performance when used in counting mode and in integrating mode. We have characterized its imaging performance by calculating the detective quantum efficiency (DQE; Rose, 1946 ▸) for both imaging modes and have used the DQE estimates to optimize the dose rate for counting. Imaging quality on actual samples was quantified by estimating the Thon rings of carbon images and by reconstructing two different cryo-EM samples to better than 3 Å resolution. Finally, the imaging modes were contrasted in terms of resolution per number of particles and per unit time by comparing the resolution of sets of apoferritin particles collected in integrating mode, integrating mode with a Volta phase plate and counting mode.

## 2. Methods   

### 2.1. Samples and grid preparation   

The apoferritin grid was provided by New York Structural Biology Center (NYSBC). The sample was prepared by placing equine spleen apoferritin from Sigma–Aldrich onto an UltrAuFoil mesh grid (Quantifoil). The grid was blotted, plunged and stored for later use.

The second grid contained *Escherichia coli* 70S ribosome cross-linked with KKL-2098 and was provided by the Dunham laboratory at Emory University. A 3 µl aliquot of ribosomes at a concentration of 100 n*M* was placed onto a plasma-cleaned UltrAuFoil grid (R1.2/1.3; Quantifoil) and vitrified using a Vitrobot Mark IV (FEI). The EM grids had been glow-discharged for 20 s using plasma cleaner (Gatan Solarus 950). The Vitrobot chamber condition was set to 100% humidity, 8°C temperature and 3.5 s blot time, and the cryogen used for vitrification was ethane-cooled with liquid nitrogen.

### 2.2. Data collection   

All data were collected with a DE64 direct electron detector in conjunction with an FEI Titan Krios microscope set to a voltage of 300 kV using *Leginon* (Suloway *et al.*, 2005 ▸). The apoferritin data were collected using counting mode, integrating mode and integrating mode with a Volta phase plate (VPP). In counting mode, the counting algorithm hardware-bins the sensor by two, resulting in a 4k × 4k sensor and doubling the apparent physical pixel size. During collection we used a magnification of 75 000× with a calibrated pixel size of 0.928 Å. The total dose for each exposure was 40 e^−^ Å^−2^ collected at a dose rate of 2.2 e^−^ Å^−2^ s^−1^ and a random defocus ranging from 0.5 to 3.0 µm. Raw frames were collected at a rate of 141 frames per second and were ‘quantized’ (grouped) into movie frames containing an average of 1 e^−^ per pixel. In integrating mode, the 8k × 8k sensor was left unbinned. During collection we used a magnification of 37 000× with a calibrated pixel size of 0.973 Å. Exposures were taken with a defocus ranging from 1.0 to 2.5 µm and a total electron dose of 61 e^−^ Å^−2^. Frames were collected at a rate of 32 frames per second with a dose rate of 50.6 e^−^ Å^−2^ s^−1^. Similarly to integrating mode, we used a magnification of 37 000× with a calibrated pixel size of 0.973 Å and a frame rate of 32 frames per second with the VPP. In this case we used a smaller defocus ranging from 0.5 to 0.75 µm with an electron dose of 30 e^−^ Å^−2^. With the VPP we used a dose rate of 7 e^−^ Å^−2^ s^−1^ for an optimal plate-charging rate. The data for *E. coli* 70S ribosome cross-linked with KKL-2098 were collected in counting mode at a dose rate of 1.3 e^−^ Å^−2^ s^−1^. We used a magnification of 59 000× with a calibrated pixel size of 1.19 e^−^ Å^−2^. A total electron dose of 25 e^−^ Å^−2^ and a defocus ranging from 1.5 to 3.0 µm were also used.

### 2.3. Data processing   

During data acquisition, micrographs were simultaneously processed using *Appion* (Lander *et al.*, 2009 ▸). Movie frames were aligned using *MotionCor*2 (Zheng *et al.*, 2017 ▸). *Gctf* v.1.06 (Zhang, 2016 ▸) and *CTFFIND*4 (Rohou & Grigorieff, 2015 ▸) were used to estimate the CTF of the aligned micrographs. Particles were template picked from the aligned micrographs with *FindEM* (Roseman, 2004 ▸). We collected 574 micrographs from the apoferritin sample using counting mode. Discriminating based on CTF confidence, 129 140 particles were extracted from micrographs with CTFs better than or equal to 4 Å resolution at the 0.5 cross-correlation coefficient mark. We collected 133 micrographs using integrating mode. Similarly, we extracted 127 681 particles from micrographs with CTFs better than or equal to 6.5 Å resolution at the 0.5 cross-correlation coefficient mark. When using integrating mode with the VPP, we collected 222 micrographs, extracting 260 063 particles. All particles were extracted using 224-pixel boxes corresponding to approximately 1.5 times the size of the particles.

All refinements of the apoferritin data were performed using *RELION*-3 with GPU acceleration. To prevent biasing the resolution of the reconstructions, we did not perform any 2D classification or 3D classification. Our first step was an initial reconstruction using all of the particles. The initial model was created by downloading the final structure from EMPIAR-10026 and low-pass filtering it to a resolution of 20 Å. Other parameters used during the refinement process were the enforcement of octahedral symmetry and a mask diameter of 142 Å. After refinement, a custom mask was created using *RELION*-3; this was expanded by five voxels with a seven-voxel Gaussian edge. The reconstructed map was sharpened using the post-processing function of *RELION*-3. Lastly, beam-tilt estimation and correction were performed within *RELION*-3 CTF refinement (Zivanov *et al.*, 2018 ▸). We continued the series of correction until no improvement was seen. The reconstructed maps of apoferritin in counting mode, integrating mode and integrating mode with the VPP were deposited in the EMDB as entries EMD-20155, EMD-20156 and EMD-20157, respectively.

For the ribosome sample, we collected 1752 micrographs. Particles were extracted from all micrographs with a box size of 384 pixels, creating a stack of 373 845 particles. The particles were initially processed with *cisTEM* (Grant *et al.*, 2018 ▸). A 2D classification of the particles was performed, only selecting classes showing defined features. The classified particles were refined and further classified using 3D classification. A final reconstruction was performed with *RELION*-3. Within *RELION*-3, beam-tilt estimations and corrections were performed until no improvement was seen. The map was deposited in the EMDB as entry EMD-20158.

## 3. Results and discussion   

### 3.1. Detector performance evaluated using NPS, MTF and DQE   

**Figure 1:**
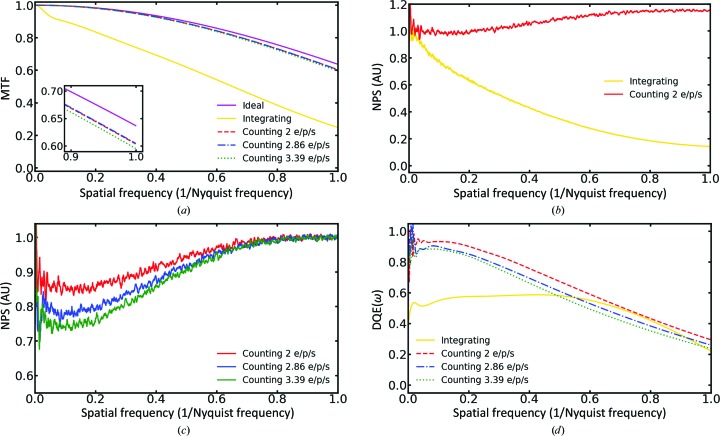
(*a*) MTF curves for the DE64 in counting mode at different dose rates, in integrating mode and the theoretical ideal curve. The inset is an enlarged view near the Nyquist frequency. (*b*) DE64 NPS curve comparison between integration and counting modes. (*c*) NPS curves for the DE64 in counting mode at different dose rates. The NPS was normalized to 1 for each dose rate. To reduce the noise in the NPS curves, a small moving average was used to smoothen the curves. (*d*) DQE curve of the DE64 in counting mode at different dose rates and in integrating mode, where ω is the spatial frequency. The DQE curves were smoothed using a cubic spline function. Dose rates are given in e^−^ per pixel per second (e/p/s).

The standard method to quantify the imaging power of a detector is to calculate its DQE, which is defined as DQE(ω) = SNR^2^_out_/SNR^2^_in_, where ω is the spatial frequency. These are difficult quantities to measure experimentally, so DQE can be reformulated in terms of the modulation transfer function (MTF) of the camera and its noise power spectrum (NPS) using the equation DQE(ω) = DQE(0) × MTF^2^(ω)/NPS_normalized_(ω) (McMullan *et al.*, 2009 ▸). Thus, to calculate the DQE values for the DE64, we first calculated its MTF using *FindDQE* (Ruskin *et al.*, 2013 ▸) and pairs of images consisting of a flat-field image with the beam blocker inserted halfway and a second image without the beam blocker inserted [Fig. 1 ▸(*a*), Supplementary Fig. S1]. When the MTFs for integrating and counting modes were compared, two different behaviors were observed. In integrating mode, the MTF decreased nearly linearly with increasing spatial frequency. The counting-mode MTF, on the other hand, was close to the ideal MTF [Fig. 1 ▸(*a*), purple]. The NPS for the different modes were calculated using an in-house Python script. Images of the beam without sample were Fourier transformed, squared and radially averaged to produce a one-dimensional NPS curve. The zero-frequency NPS was normalized to 1 as described in McMullan *et al.* (2009 ▸) [Fig. 1 ▸(*b*)]. Since counting detectors binarize individual electron hits, they are sensitive to the electron-dose rate and are susceptible to coincidence loss (Li *et al.*, 2013 ▸). Coincidence loss occurs when multiple electron hits are recorded as one owing to their coincidence in position or time. Coincidence loss is reflected in the NPS as a suppression of the low-frequency power [Fig. 1 ▸(*c*)]. The DE64 in counting mode operates at a frame rate of 141 frames per second and NPS were calculated with dose rates of 2, 2.86 and 3.39 e^−^ per pixel per second. There was a clear trend where 3.39 e^−^ per pixel per second produced a substantial depression of the power at NPS(0). The depression was lessened at 2 and 2.86 e^−^ per pixel per second. We did not try lower dose rates as this would have required unreasonably long exposures. The final value required for the DQE calculation, DQE(0), was calculated as described in McMullan *et al.* (2014 ▸), where DQE(0) ≃ 1 − (9/2)*n* − (239/12)*n* ^2^ and *n* is the average electrons per frame per pixel. Given the MTF, NPS and DQE(0), DQE curves were calculated for both integrating and counting modes, demonstrating that they both show good performance over a broad range of frequencies. Both modes have similar values near the Nyquist frequency but differ at low frequencies, where the counting mode starts at a value of 0.93 and the integrating mode starts at a value of 0.52. In this way the DE64 is unique, since other studies have shown that the DQEs at high frequencies for integrating modes on the Falcon III (Song *et al.*, 2019 ▸) and Gatan K2 (Chang *et al.*, 2016 ▸) were substantially lower than for their counting modes. When comparing the DE64 in counting mode with the past generation of DEDs, the DE64 demonstrates a superior MTF and DQE. In Supplementary Fig. S4, the MTF and DQE curves of the DE64 are compared with those of the Gatan K2 and DE20. The Gatan K2 plots were obtained from the Grigorrieff laboratory website, where they are publicly available, and the DE20 plots are from images that we had collected in the past (Spear *et al.*, 2015 ▸). The MTFs of the DE64 and DE20 started at 1, while that of the K2 started slightly lower. Towards higher frequencies, the DE20 MTF declined faster than those of the DE64 and K2. Ultimately, the DE64 had a superior MTF to the DE20 and K2 over all frequencies. As reported in Ruskin *et al.* (2013 ▸), the DQE starting value for the Gatan K2 is 0.81 and that for the DE64 in counting mode is 0.88. The difference between the DQEs appears to be reduced at higher frequencies, but nonetheless the DE64 continued to have a higher DQE all the way to the Nyquist frequency.

### 3.2. Comparing contrast transfer functions   

An important step for cryo-EM reconstruction is the accurate determination of the contrast transfer function (CTF). The CTF oscillations of an image not only increase with resolution, but the amplitude of the oscillations is also dampened. This dampening makes it difficult to properly correct the CTF at high frequencies, limiting the useful information contained in an image. For this reason, it is beneficial to conserve as much power of the CTF as possible. In Supplementary Fig. S2, we present the Thon rings and CTF fitting of two amorphous carbon images collected in integrating mode and in counting mode. The upper two panels [Supplementary Figs. S2(*a*) and S2(*b*)] correspond to integrating mode, while the bottom two [Supplementary Figs. S2(*c*) and S2(*d*)] correspond to counting mode. In both images the Thon rings [Supplementary Figs. S2(*a*) and S2(*c*)] extend past the 4 Å resolution mark and continue out to the Nyquist frequency. The CTF of both images was fitted past 3 Å resolution with a cross-correlation coefficient (CC) of ≥0.8. Although the CTF can be fitted to high resolution in both modes, counting mode contains better power across all frequencies and is especially noticeable at lower frequencies, as seen in Supplementary Figs. S2(*b*) and S2(*d*), reflecting the results of the DQE.

### 3.3. High-resolution apoferritin and 70S ribosome structures   

**Figure 2:**
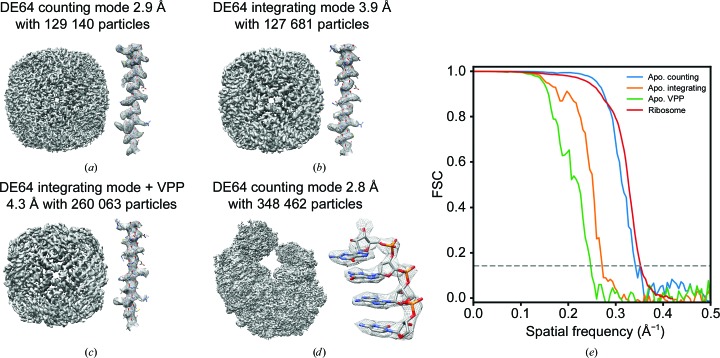
(*a*) Counting-mode density map with a resolution of 2.9 Å using 129 140 particles. (*b*) Integration-mode density map with a resolution of 3.9 Å using 127 681 particles. (*c*) Integration mode using a Volta phase plate density map with a resolution of 4.3 Å using 260 063 particles. (*d*) Counting-mode density map with a resolution of 2.8 Å using 348 462 particles. (*e*) Fourier shell correlation of all reconstructions.

Since the resolution of cryo-EM reconstructions depends on both the quality and the quantity of the images, the question arises whether it is better to collect more data in integrating mode or better data in counting mode. We compared the camera modes by collecting and reconstructing data from the protein complex apoferritin, as it can only be reconstructed with high-quality images (Henderson & McMullan, 2013 ▸; Russo & Passmore, 2014 ▸; Massover, 1993 ▸). In addition to the two camera modes, we also used integrating mode together with a Volta phase plate (VPP), which boosts low-frequency contrast. The idea here is that a combination of the boosted low-frequency contrast from the VPP with the increased throughput for integrating mode might produce a better result than the counting or integrating modes alone. We collected a total of 148 788 particles for counting mode, 152 220 particles for integrating mode and 264 784 particles for integrating mode with the VPP (representative images are shown in Supplementary Fig. S5). These were refined using *RELION*, resulting in reconstructions with resolutions of 2.9 Å for counting mode (EMD-20155), 3.9 Å for integrating mode (EMD-20156) and 4.3 Å for integrating mode with the VPP (EMD-20157), as seen in Fig. 2 ▸. Clearly, despite the lower throughput, counting mode boosted the resolution that we were able to reach with approximately the same number of particles when compared with integrating mode. The increased low-frequency contrast from the VPP did not help the resolution in integrating mode even with increased numbers of particles. Given that counting mode produced the best resolution on a per-particle basis, we also characterized the performance of counting mode with an asymmetric particle. We collected 373 845 particles from a 70S ribosome sample. This was classified down to 348 462 particles and refined in *RELION*. The resulting reconstruction achieved a resolution of 2.8 Å (EMD-20158) with well resolved features for the RNA [Fig. 2 ▸(*d*)]. Notably, this resolution was at ∼4/5 of the Nyquist frequency, indicating that the resolution was beginning to be limited by sampling and that higher resolution could likely be achieved by increasing the magnification during data collection.

### 3.4. Resolution versus throughput   

**Figure 3:**
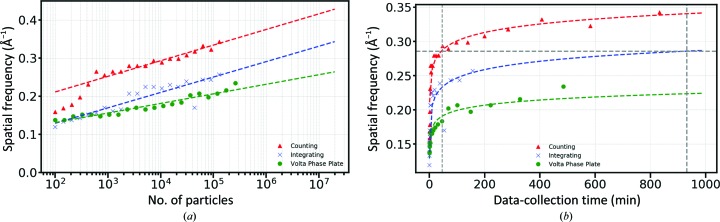
(*a*) ResLog plots for apoferritin collected in counting, integrating and integrating with VPP modes, respectively. (*b*) Plot of resolution against time of data collection. The horizontal line is the 3.5 Å resolution mark. The vertical lines are at 46 and 931 min.

The preceding data clearly indicate that data quality is critical for obtaining high resolution, but since resolution is dependent upon the number of particles contributing to a reconstruction (LeBarron *et al.*, 2008 ▸; Rosenthal & Henderson, 2003 ▸), data-collection throughput is also a critical parameter. It has been shown that to a first approximation there is a linear relationship between spatial frequency and the logarithm of the number of particles contributing to a reconstruction. These can be plotted to generate a ResLog plot (Stagg *et al.*, 2014 ▸), in which the *y* intercept of the plot is related to the alignability of a given data set and the slope is related to the quality of the imaging. We performed a ResLog analysis for each of the three methods of data collection to understand how the reconstruction resolutions evolve as more particles are collected. This resulted in several observations about the quality of the data for the three different modes of data collection that we used. We expected the VPP integrating particles to be more alignable than integrating alone given the boost in low frequency when the VPP is used. However, the ResLog plots in Fig. 3 ▸(*a*) showed that they both share essentially the same *y* intercept, indicating that the particles in both data sets are equally alignable. The ResLog slope, however, was lower for VPP integrating than it was for integrating alone. It is unclear why this may be, but we speculate that this is owing to the additional challenges of estimating the phase shift as well as the defocus when using the VPP. It is possible that inaccuracies in these estimates are what was limiting the per-particle improvement in resolution for this mode. The counting-mode particles had the highest *y* intercept, indicating the best alignability of all particles. Interestingly, integrating and counting modes have similar slopes, and as a result the resolution of both increases at the same rate. It should be noted that counting-mode resolution outperforms integrating-mode resolution whether we look at it in terms of number of particles or in terms of data-collection time.

The different modes of data collection with the DE64 have substantial differences in their data-acquisition times. Integrating mode has the highest throughput, and the exposure time for 61 e^−^ Å^−2^ is 1.3 s. It also uses the full 8k × 8k sensor. In contrast, the exposure time for 40 e^−^ Å^−2^ in counting mode is 20.8 s and the counting algorithm for the DE64 hardware-bins the sensor by 2, resulting in a 4k × 4k image. The acquisition time for a 30 e^−^ Å^−2^ VPP integrating-mode image is 4.6 s. In real data collections with apoferritin, we obtained 42 micrographs per hour in integrating mode when using one exposure per focus, resulting in 49 178 particles per hour. This can be increased to 113 micrographs or 132 312 particles per hour if we move to four exposures per focus (Cheng *et al.*, 2018 ▸) using image shift. In contrast, counting mode is limited to 36 micrographs or 9349 particles per hour. The Volta phase plate is limited to only a single exposure per focus since using image shift would result in an asymmetric buildup of charge on the VPP owing to beam tilt. Acquisition with the VPP in integrating mode produced 27 micrographs or 32 203 particles per hour. When the resolution was plotted against the time that it took to acquire the particles in the different acquisition modes, counting mode was clearly superior in terms of resolution improvement over time. Remarkably, as seen in Fig. 3 ▸(*b*), it would take 15.5 h to collect sufficient particles to achieve 3.5 Å resolution in integrating mode, while this resolution could be achieved in 46 min with particles collected in counting mode. Thus, the speed advantages of integrating mode are dwarfed by the increases in image quality in counting mode.

### 3.5. Frame tracking   

Another notable difference between the camera modes is the tracking of the frame alignment, as seen in Supplementary Fig. S3. The integrating-mode frame alignment shows a displacement of about 1 Å or about a single pixel. We suspect that the exposure time of integrating mode is so short that the particles do not have enough time to move. On the other hand, the counting-mode frames show a total displacement of 23 Å. Although counting-mode particles experience a lot of movement, the frame-alignment software is capable of aligning the frames with a high degree of accuracy, presumably owing to the high DQE at low frequencies. The high-resolution maps that are achieved after frame alignment seem to support this assumption.

## 4. Conclusions   

Here, we have shown that counting mode delivers higher resolution cryo-EM reconstructions than integrating mode, regardless of the number of particles contributing to the maps or the length of data collection. The throughput of integrating mode was over ten times greater than that of counting mode, but the superior low-frequency DQE of counting mode resulted in better resolution 3D reconstructions per unit of time spent collecting data. Given the high cost of data-collection time on a high-end cryo-EM microscope, these results are significant for deciding how to collect data for a given sample. Integrating mode allows the collection of large numbers of particles in a short period of time, and this can be useful for samples that are easily alignable and have high symmetry. For more challenging samples, it is clear that counting mode is the better choice. Given our results, we suspect that the high DQE of counting mode for the DE64 will enable the structure determination of even more challenging single-particle projects, particularly those with low symmetry and with masses of less than 150 kDa.

## Supplementary Material

EMDB reference: apoferritin, EMD-20155

EMDB reference: EMD-20156

EMDB reference: EMD-20157

EMDB reference: 70S ribosome, EMD-20158

Supplementary Figures. DOI: 10.1107/S2052252519012661/eh5003sup1.pdf
