# Supplementary material for: Throughput and resolution with a next-generation direct electron detector
Source: IUCrJ. 2019 Oct 24;6(Pt 6):1007–13. doi: 10.1107/S2052252519012661 (PMC6830211; doi:10.1107/S2052252519012661)
Supplement: Supplementary file 1 [file m-06-01007-sup1.pdf]

IUCrJ

Volume 6 (2019)

Supporting information for article:

Throughput and resolution with a next-generation direct electron detector

Joshua H. Mendez, Atousa Mehrani, Peter Randolph and Scott Stagg

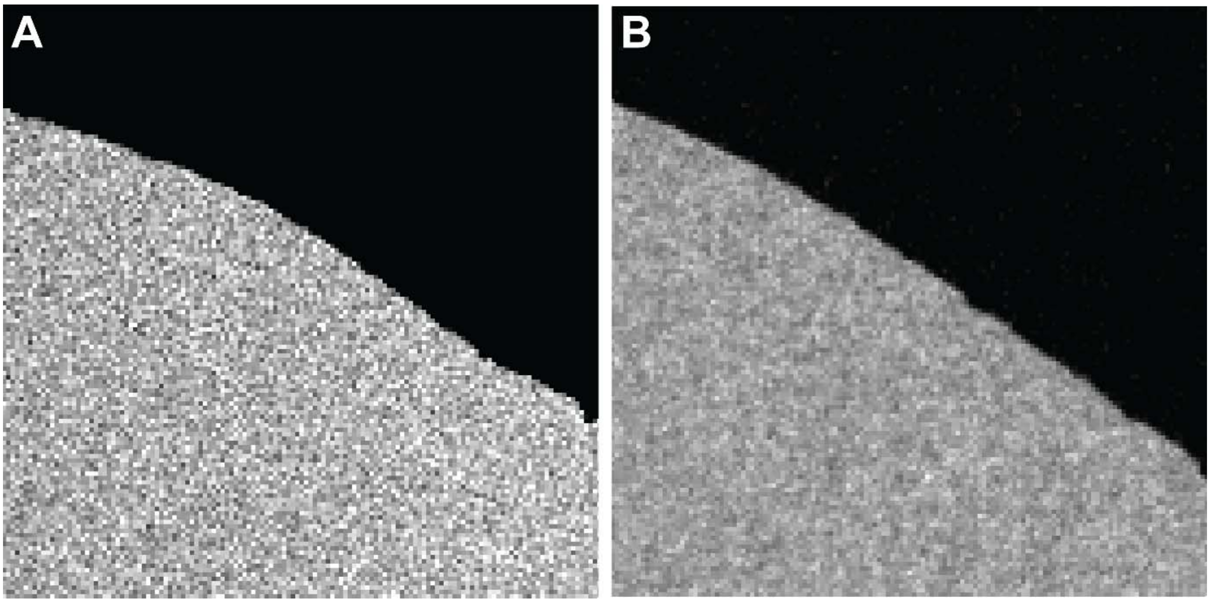

Figure S1 A) Beam stop sharp edge imaged in counting mode. B) Beam stop sharp edge imaged in integrating mode

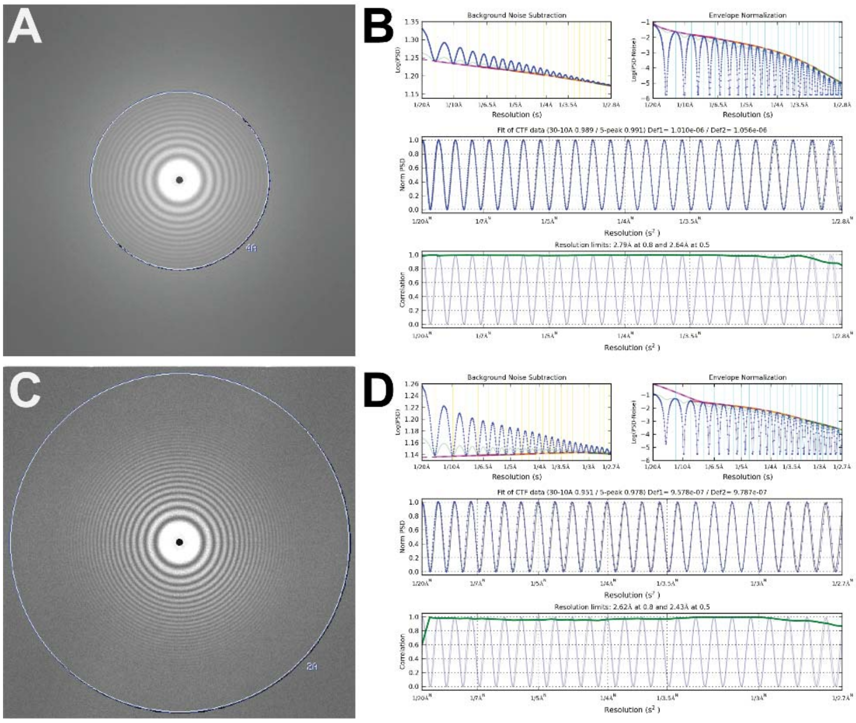

Figure S2 A) Thon rings of carbon from an image acquired using DE 64 in integration mode. B) 1-dimension radial average of the power spectrum with the fitted contrast transfer function (CTF) as seen in Appion. C) Thon rings of carbon from an image acquired using DE 64 in counting mode. D) 1-dimension radial average of the power spectrum with the fitted CTF as seen in Appion.

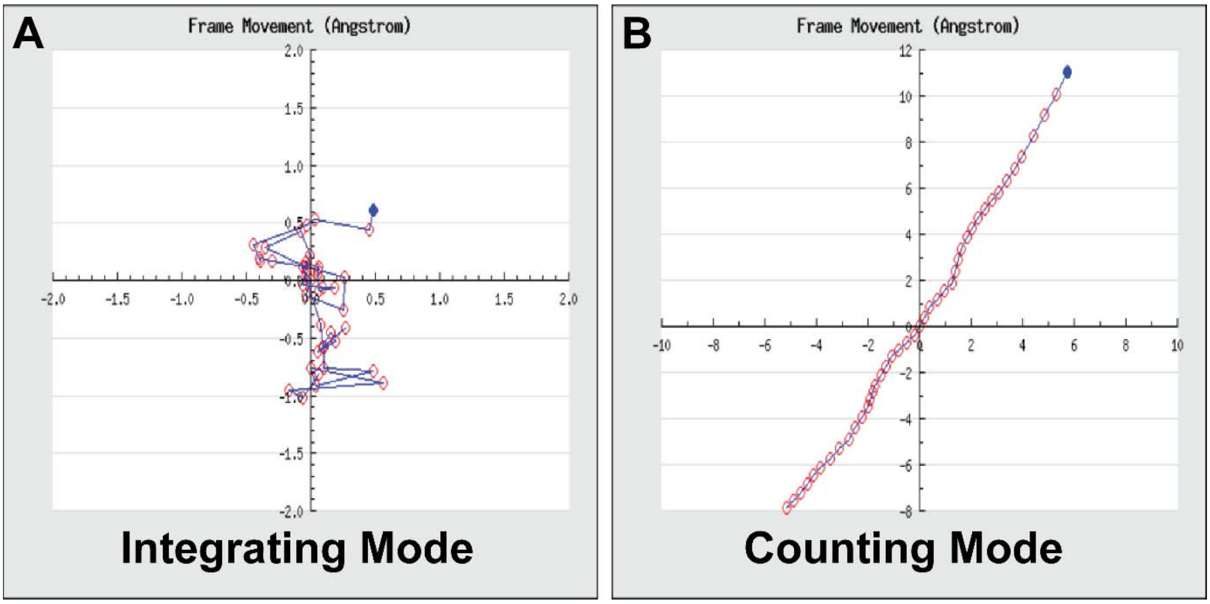

Figure S3 Frame movement tracking as seen in Appion. A) Frame movement of data collected in integrating mode. B) Frame movement of data collected in counting mode.

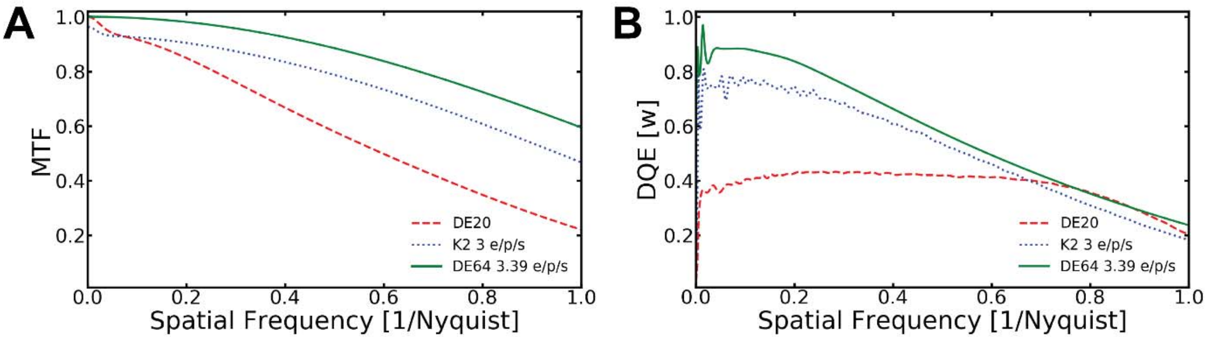

Figure S4 A) MTF curve of Gatan K2 at 300 kV (obtained from Grigorieff Lab website) with dose rate of 3 e^-/p/s, DE20 and DE64 in counting mode with dose rate of 3.39 e^-/p/s. B) Corresponding DQE curves for the Gatan K2, DE20 and DE64.

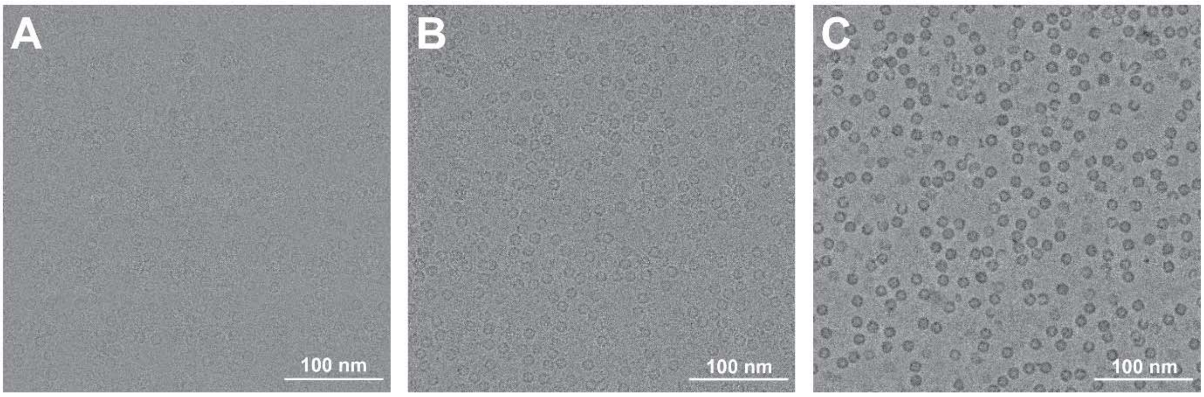

Figure S5 A) Micrograph collected in counting mode. B) Micrograph collected in integrating mode. C) Micrograph collected in integrating mode with VPP.
